# Radiomics Applications in Renal Tumor Assessment: A Comprehensive Review of the Literature

**DOI:** 10.3390/cancers12061387

**Published:** 2020-05-28

**Authors:** Rodrigo Suarez-Ibarrola, Mario Basulto-Martinez, Alexander Heinze, Christian Gratzke, Arkadiusz Miernik

**Affiliations:** 1 Department of Urology, Faculty of Medicine, University of Freiburg Medical Centre, 79106 Freiburg, Germany; christian.gratzke@uniklinik-freiburg.de (C.G.); arkadiusz.miernik@uniklinik-freiburg.de (A.M.); 2 Department of Urology, Hospital Regional de Alta Especialidad de la Peninsula de Yucatan, Merida 97133, Mexico; basultourologia@gmail.com; 3 Department of Urology, Marienkrankenhaus, 22087 Hamburg, Germany; heinze01@gmail.com

**Keywords:** radiomics, texture analysis, machine learning, deep learning, artificial neural network, small renal mass, angiomyolipoma, oncocytoma, renal cell carcinoma, kidney cancer

## Abstract

Radiomics texture analysis offers objective image information that could otherwise not be obtained by radiologists′ subjective radiological interpretation. We investigated radiomics applications in renal tumor assessment and provide a comprehensive review. A detailed search of original articles was performed using the PubMed-MEDLINE database until 20 March 2020 to identify English literature relevant to radiomics applications in renal tumor assessment. In total, 42 articles were included in the analysis and divided into four main categories: renal mass differentiation, nuclear grade prediction, gene expression-based molecular signatures, and patient outcome prediction. The main area of research involves accurately differentiating benign and malignant renal masses, specifically between renal cell carcinoma (RCC) subtypes and from angiomyolipoma without visible fat and oncocytoma. Nuclear grade prediction may enhance proper patient selection for risk-stratified treatment. Radiomics-predicted gene mutations may serve as surrogate biomarkers for high-risk disease, while predicting patients’ responses to targeted therapies and their outcomes will help develop personalized treatment algorithms. Studies generally reported the superiority of radiomics over expert radiological interpretation. Radiomics provides an alternative to subjective image interpretation for improving renal tumor diagnostic accuracy. Further incorporation of clinical and imaging data into radiomics algorithms will augment tumor prediction accuracy and enhance individualized medicine.

## 1. Introduction

In 2018, renal cell carcinoma (RCC) accounted for 403,300 newly diagnosed cancer cases and 175,100 deaths worldwide [1]. In the United States alone, RCC is the sixth most common cancer in men and eighth in women, accounting for 5% and 3% of all newly diagnosed cases annually, respectively [2]. An increasing incidental detection of renal masses with cross-sectional imaging led to the diagnosis of more asymptomatic, small, and clinically localized renal masses. Small renal masses (SRMs), defined as ≤4 cm in diameter, account for more than 50% of all renal masses, approximately 10–30% of which result in benign histology [3,4]. The diagnosis of SRMs carries the risk of subjecting patients to unnecessary procedures and overtreating lesions that may not progress.

Renal tumor biopsy (RTB) provides a means for tissue sampling to assist in tumor histological and subtype diagnosis for risk-stratified management [4]. Although it shows high diagnostic accuracy for RCC, RTB is an invasive procedure and it is criticized for its inability to sample tumors at multiple sites and distinguish tumor histologic subtypes and nuclear grade [5]. Due to its improved soft-tissue contrast, magnetic resonance imaging (MRI) outperforms computed tomography (CT) in the evaluation of indeterminate renal masses [6], local invasion, and intravascular extension [7]. However, the differentiation of benign lesions, particularly oncocytoma and angiomyolipoma without visible fat (AMLwvf), from RCC can be challenging due to subjective radiological image interpretation [8]. Radiomics is a term that encompasses various techniques for the extraction of quantitative features from medical images to improve diagnostic, prognostic, and predictive image interpretation accuracy. It is essentially the conversion of images into metadata for subsequent mining to improve clinical decision-making algorithms [9]. It assists physicians in identifying complex image patterns that are not visible to the naked eye by using artificial intelligence (AI) methods [10]. Recent advancements in AI, specifically in machine and deep learning, accelerated the application of radiomics to medical imaging as a new beacon to guide clinical decisions.

## 2. Materials and Methods

### 2.1. Study Aims

The primary objective was to perform a comprehensive review of the literature on current radiomics applications in renal mass assessment. The secondary objective was to provide a narrative summary of articles evaluating the accuracy of radiomics for distinguishing benign and malignant tumors, predicting nuclear grade, obtaining gene expression-based biomarkers, and prognosticating RCC patients.

### 2.2. Literature Search

A comprehensive search of English language literature was conducted using the PubMed-MEDLINE database up to 20 March 2020. To capture recent trends in radiomics applications, the search was limited to articles published within the last five years. The search strategy included the following broad terms in isolation or combination: “kidney cancer”, “renal cell carcinoma”, “machine learning”, “deep learning”, and “radiomics”. We repeated searches on all newly identified articles until no further relevant articles were found. Titles and abstracts of articles that were identified by the keyword search were retrieved for full-text evaluation. Potentially eligible articles were independently screened against the study selection criteria by two authors (R.S. and M.B.).

### 2.3. Inclusion and Exclusion Criteria

**Figure 1:**
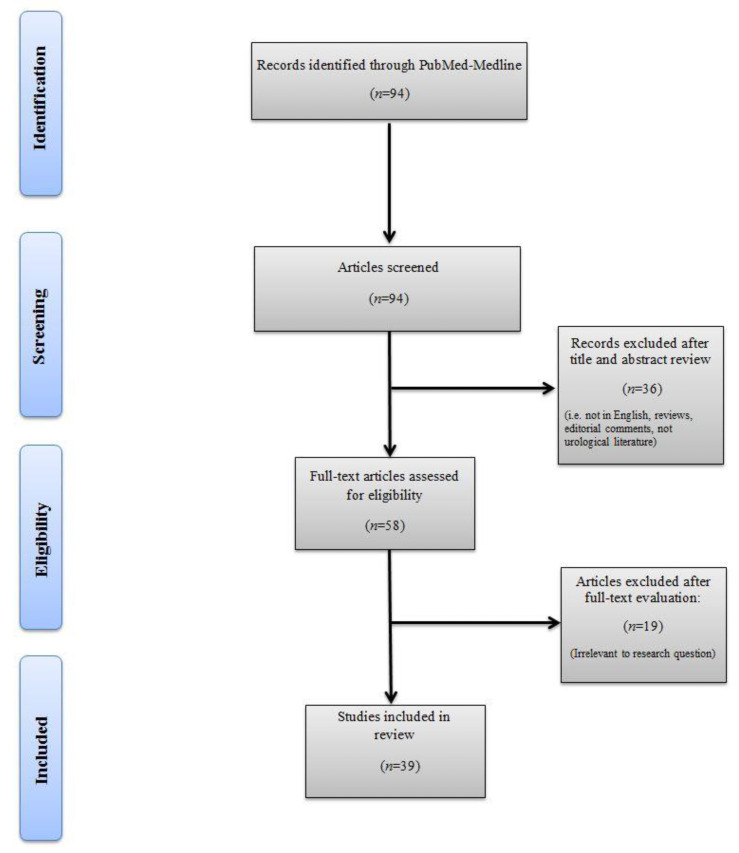
Summary of study selection process.

Two authors (R.S. and M.B.) individually determined inclusion/exclusion of all articles retrieved in full text, and discrepancies were resolved through discussion by a third reviewer (A.M.). Studies that met the following criteria were included: (a) renal tumor radiomics-based analysis; (b) articles written in English; (c) peer-reviewed publications; (d) methodology documented in replicable detail. After article selection and according to the eligibility criteria, the following types of studies were excluded: articles not related to renal tumors, articles not written in English, review articles, editorials, and replies from author. In accordance with the Preferred Reporting Items for Systematic Reviews and Meta-Analyses (PRISMA) criteria, Figure 1 was included to delineate our article selection process. After full-text evaluation, data were independently extracted by the authors for further assessment of qualitative and quantitative evidence synthesis.

### 2.4. Data Extraction and Quality Assessment

Data extraction from each study was independently conducted by two authors (R.S. and M.B.). As proposed by the PRISMA guidelines, we used the population, intervention, comparator, outcomes, and study design approach to specify the eligibility criteria. The studies were considered eligible if patients with renal masses (population) were evaluated with CT/MRI/positron-emission tomography (PET)-based radiomics data (intervention), compared to radiologists’ subjective image assessment (comparator) or as a single-arm study group, to investigate the accuracy for benign and malignant tumor differentiation, to predict nuclear grade, identify molecular biomarkers, and determine patient outcomes (outcome). After full-text evaluation, data were independently extracted by the authors for further assessment of qualitative and quantitative evidence synthesis. The following information was extracted from each study: name of author, journal and year of publication, imaging method, number of patients per study, mean age, mean lesion diameter, radiomics method, texture features extracted, and outcome prediction accuracy.

## 3. Results

### 3.1. Characteristics of Included Studies

A total of 94 articles were identified from the search query. Overall, 36 reports were excluded after title and abstract review, for the following reasons: 10 not related to urology, 16 not related to renal cell carcinoma, five review articles, four articles not in English, and one reply to authors. After full-text evaluation, 19 articles were excluded because they did not meet the study´s objectives. Finally, we carefully read the full text of each of the remaining 39 articles which were included in the review. The flow chart of the study selection process is shown in Figure 1. The 39 included studies were divided into four main categories according to study objectives: 19 for tumor differentiation, 11 for nuclear grade prediction, six for gene expression-based molecular biomarkers, and three for patient outcome prediction. Tumor differentiation was further subclassified into four for malignant vs. benign renal mass diagnosis, seven for RCC vs. angiomyolipoma without visible fat, four for RCC vs. oncocytoma, and four for RCC subtypes.

### 3.2. Renal Mass Differentiation

The adoption and widespread use of cross-sectional imaging increased the detection rate of renal masses [11]. However, the diagnostic migration toward early disease stages was not translated into improved cancer-specific mortality for localized RCC, indicating that many incidental renal masses are unlikely to progress and pose a threat to patients in the long term [12]. These findings contributed to the growing awareness in the overdiagnosis and overtreatment of these tumors.

The decision whether to treat patients with renal masses is often made without a definitive histologic diagnosis. Therefore, treatment decision-making and management are heavily reliant on clinical imaging. Although RTB can be used to differentiate malignant from benign histology, its role is selective and limited in its ability to discern between aggressive and indolent disease. Several studies showed an inverse correlation between renal mass radiographic size and the probability of harboring benign or low-grade pathology in surgical specimens [13]. Although the likelihood of malignancy increases with mass size, current imaging modalities and subjective radiological image interpretation cannot reliably distinguish malignant tumors from certain benign tumors, such as oncocytomas and AMLwvf [14].

Studies showed that renal masses including RCC subtypes, AMLwvf, and oncocytomas can be portrayed with distinct gray-level imaging patterns that are traceable by radiomic analysis [15]. Radiomics includes a number of approaches designed to convert medical images to quantitative, minable, and high-dimensional data [3]. Machine (ML) and deep learning (DL) algorithms are used to automatically extract and analyze histogram, texture, and shape information from imaging data which may not be evident to the naked eye. Given the limitations of conventional medical imaging, there is increased interest to apply radiomics in oncological imaging as a tool to obtain diagnostic, predictive, and prognostic information from routine clinical imaging [16]. However, despite its extensive use in research and favorable results linking CT/MRI texture features to renal mass characterization, the routine use of radiomics in clinical practice is yet to be seen. For imaging markers, including texture-based metrics, to bridge the translational gap between an experimental research tool and a clinically applicable diagnostic algorithm, its technical and biological validity, biological validity, qualification, and cost-effectiveness need to firstly be established [3].

#### 3.2.1. Benign vs. Malignant Renal Masses

Kunapuli et al. compared 11 ML methods for the development of a clinical decision support (CDS) tool to classify renal masses. Features characterizing relevant metrics were extracted from four-phasic contrast enhanced computed tomography (CECT) images of 150 patients with various benign and malignant lesions. Overall, their relational functional grading boosting (RFGB) model achieved the highest prediction accuracy and area under the curve (AUC) (0.83) from all 11 methods and was identified as a promising CDS tool for renal mass identification [17]. Similarly, Erdim et al. compared eight ML algorithms to construct a prediction model for renal mass diagnosis based on CECT imaging from both benign and malignant lesions. The random forest model showed the highest accuracy (90.5%) and AUC (0.92) and was a suitable method to distinguish benign and solid renal masses in nine-tenths of patients [18]. Sun et al. compared the performance of radiologic radiomic ML models and expert-level radiologists to differentiate benign from malignant solid renal masses using CECT examinations. The radiomic ML model yielded overall higher performance values in terms of sensitivity and specificity for differentiating clear cell renal cell carcinoma (ccRCC) from papillary RCC (pRCC) and chromophobe RCC (chRCC), ccRCC from AMLwvf and oncocytoma, and pRCC and chRCC from AMLwvf and oncocytoma [15]. Similarly, Xi et al. developed a DL model based on MRI data and compared it to four expert radiologists to distinguish benign renal tumors from RCC. The DL model showed overall higher accuracy, sensitivity, and specificity and was comparable to expert diagnostic opinion [19]. Table 1 summarizes articles investigating the differentiation of benign from malignant lesions using radiomics strategies.

#### 3.2.2. Angiomyolipoma (AMLwvf) vs. RCC Subtypes

Angiomyolipoma (AML), the most common benign solid renal tumor, is composed of varying amounts of dysmorphic blood vessels, smooth muscle components, and mature adipose tissue [35]. Its diagnosis relies on the identification of macroscopic fat that is normally easily detected by CECT or MRI. However, up to 5% of AMLs lack macroscopic fat, a condition known as AML without visible fat (AMLwvf), making it challenging to differentiate them from RCC by conventional imaging [36]. Several studies described certain imaging features that are highly suggestive of AMLwvf and analyzed them with radiomics models [20,21,22,23,25,26].

By applying artificial neural network (ANN) classifiers, Yan et al. showed that texture analysis (TA) may be a reliable quantitative strategy to differentiate between AMLwvf, ccRCC, and pRCC with an accuracy in the range of 90.7–100% based on three-phasic CECT scan images [20]. Other investigations employed similar strategies, although with ML-based TA from CECT images, and they reported higher accuracy (93.9%) and AUC (0.955) [21,24]. Moreover, Cui et al. proposed an automatic computer identification system to differentiate AMLwvf from all RCC subtypes from whole-tumor CECT images using an over-sampling technique to increase the sample volume of AMLwvf [23]. They showed that morphological interpretation by radiologists achieved overall lower performance differentiating AMLwvf from all RCC subtypes. While some studies investigated deep feature classification, by using ML and ANN-based classifiers, to improve texture features to further enhance the differentiation between AMLwvf and RCC [22], others built radiomics nomograms from these texture features, showing that they outperform other models using solely clinical factors [25]. Interestingly, Ma et al. not only showed that whole-tumor radiomics-based CECT analysis is superior to conventional CECT analysis, but also that the unenhanced phase showed higher AUC than the corticomedullar phase (CMP) and nephrographic phase (NP) [26]. These findings are encouraging for the future acquisition of imaging studies without the need of contrast agents to spare patients with renal masses the associated radiation burden of multi-phasic CECT. Table 1 summarizes studies investigating the differentiation of AMLwvf from RCC subtypes.

#### 3.2.3. Oncocytoma vs. RCC Subtypes

Renal oncocytoma is a benign renal tumor, accounting for approximately 3–7% of all renal tumors [37]. Although it is widely believed that a central stellate scar is its main characteristic, it was reported present in only 46% of cases and may also appear in 26% of chRCC [38]. Moreover, there are overlapping imaging features between ccRCC and oncocytoma making their differentiation challenging [39]. Therefore, a reliable radiomics method that is non-reliant on the presence of a central scar to characterize oncocytomas would be an invaluable diagnostic tool.

Yu et al. evaluated the utility of TA for the distinction of oncocytoma from RCC subtypes. The ML model’s ability to distinguish both ccRCC and pRCC from oncocytoma was excellent with AUC of 0.93 and 0.99, respectively [27]. Li et al. explored the clinical value of radiomics-based approaches, by combining TA with five ML-based models, to differentiate oncocytoma from chRCC. They found that all five classifiers performed well at differentiating between the two with AUC values over 0.85 and concluded that their approach provides valuable preoperative diagnostic accuracy [29]. Other studies used DL approaches such as convolutional neural networks (CNN) to accurately classify chRCC and oncocytoma while also achieving 100% sensitivity in comparison with final pathology results [30]. Coy et al. investigated the diagnostic value and feasibility of a DL-based renal lesion classifier to differentiate ccRCC from oncocytoma in 179 patients with pathologically confirmed renal masses on routine four-phasic CECT [28]. When using the entire tumor volume in combination with tumor mid-slices, the excretory phase showed the best classification performance with 74.4% accuracy, 85.8% sensitivity, and a positive predictive value of 82.5%. Table 1 summarizes articles investigating the distinction between oncocytoma and RCC subtypes.

#### 3.2.4. RCC Subtype Differentiation

According to cell appearance, RCCs can be largely categorized into three major subtypes: ccRCC, pRCC, and chRCC, which constitute more than 90% of all RCCs [40]. The clear cell variant is the most lethal subtype, being most likely to metastasize, whereas the pRCC and chRCC subtypes show better survival rates [41]. Therefore, RCC subtyping is clinically important due to the increased use of novel molecular targeted therapeutic agents.

Kocak et al. developed models to distinguish between the three major RCC subtypes using ML-based quantitative CECT TA. Although texture features derived from CMP performed better than those from unenhanced phase, ML-based TA was relatively poor for distinguishing between the three major RCC subtypes with overall 69.2% accuracy. However, when using CMP images with an ANN boosting algorithm, accuracy improved to 84.6% [32]. Similarly, Han et al. exploited a DL framework to distinguish between RCC subtypes using CECT images. Three-phasic input images were fed to an ANN; its performance was tested with a dataset of 169 biopsy-proven cases and showed an AUC of 0.90 regardless of subtypes [34]. Li et al. developed ML-based radiomics models with CECT images for differentiating ccRCC from non-clear-cell variants and investigated a potential radiogenomics link between imaging features and the von Hippel–Lindau gene mutation. The eight most relevant CMP features were selected to build a ML-based model which achieved a validation AUC of 0.95 and 92.5% accuracy. Five out of the eight all-relevant features were significantly associated with the von Hippel–Lindau gene mutation [31].

Yin et al. combined positron-emission tomography (PET)/MRI-based radiomics as a surrogate biomarker for intratumoral disease risk of molecular subtype ccA and ccB in patients with primary ccRCC [33]. The sparse partial least squares discriminant analysis method was applied to 168 radiomics features selected from 23 specimens of eight patients. Using radiomics features only, the correct classification rate for molecular subtype classification was 86.96%. When combining radiomics features with clinical parameters, mRNA, and microvascular density from each specimen, the best classification rate was 95.65%.

### 3.3. Nuclear Grade Prediction

The International Society of Urological Pathology (ISUP) and Fuhrman grading systems categorize patients’ risk based on pathological features, discriminating between high- and low-risk tumors, which correlate with prognosis. The heterogeneity of tumor images quantitatively assessed by radiomics may enhance proper patient selection for surveillance avoiding RTB, and in decision-making between nephron-sparing and radical approaches. RTB carries risks of invasiveness and sampling bias; therefore, it is reasonable to search for accurate, safe, and non-invasive methods. Moreover, 16% of nuclear grade upstaging was reported on surgical pathology, resulting in an accuracy between 51.5% and 75.9% for grade discrimination [13].

Several retrospective studies evaluated the performance of radiomics for predicting RCC nuclear grade and showed satisfactory accuracy (Table 2). Two studies assessed the accuracy of non-enhanced-CT scan images to differentiate between low- and high-grade tumors. An 85.1% accuracy was reported using ML-based TA, similar to the 81.5% accuracy reported when using ANN algorithms [42,43].

Moreover, most studies built models with texture features retrieved from different phases of CECT scans. Models combining texture features from several phases outperformed those using single phase-based TA. Lin et al. used ML classifiers to extract texture features from every CT scan’s phase (pre-contrast phase, CMP, and NP) and compared each phase alone to three-phase CT images. The model combing all phases achieved the best accuracy (74%), positive predictive value (91%), negative predictive value (59%), and AUC (0.87). The top-ranked models were reported by He et al. with a predictive mean value of 92.5% ± 1.83% using ANN-based radiomics. The best accuracy (94.1% ± 1.14%) was achieved by combining texture features from conventional image which were calculated from manually selected regions of interest (ROI), such as mean attenuation, parenchyma attenuation and absolute enhance attenuation, and CMP [45,48,49,50,51].

MRI has the advantage of fine soft-tissue characterization while avoiding radiation exposure; therefore, Goyal et al. aimed to identify texture features from multiphasic MRI in a small cohort of patients (*n* = 29) and found several features that provided excellent radiomics performance, where the top three features held AUCs >0.82 [47]. Likewise, Cui et al. performed TA from multiphasic CECTs (*n* = 347) and MRIs (*n* = 93) with an external validation of patients (*n* = 20) who underwent both modalities. Interestingly, ML classifiers based on all-sequence MRI images (71% to 73% in internal and 64% to 74% in external validation) and all-phase CT images (77% to 79% in internal and 61% to 69% in external validation) had significant increases in accuracy [52].

The creation of risk assessment scores for personalized medicine is promising, and radiomics is a step forward in such a pathway by adding TA-based scores that might improve prediction accuracy. A predictive score beyond textures features was reported by Ding et al., integrating ML-based texture and non-texture features. Overall, a round-shape was a good discriminator of high-grade tumor (AUC: 0.723 (0.632–0.803)) similar to TA features; however, a model including both texture and non-texture features did not outperform the TA-based model’s accuracy [44].

Similarly, image TA beyond the tumor was investigated by Gill et al. who compared TA from juxtatumoral perinephric fat between low- and high-grade tumors and found that the majority of features were significantly different, and the gray-level co-occurrence matrix had the best accuracy (AUC 0.746 (0.63–0.86)) [46].

The clinical utility of radiomics to discriminate between low- and high-grade tumors is yet to be determined; however, high accuracies were reported especially when using multi-phase images. MRI was not proven to be better for discrimination, but this was based only on small patient samples. Moreover, including texture feature analysis from tissues beyond the tumor and adding other quantitative data based on the standard radiological features commonly used might improve radiomics performance. Unifying radiomics to personalized medicine algorithms using clinical features and even genomic data might help build more robust prediction scores.

### 3.4. Gene Expression-Based Molecular Biomarkers

Predicting molecular biomarkers by radiomics entails potential clinical utility, as TA-derived scores might achieve appropriate accuracy and serve as surrogate biomarkers. Studies addressing this topic are enlisted in Table 3. 

*BRCA1*-associated protein (*BAP1*) gene mutations are found in ~15% of ccRCC and are associated with high-grade tumors and poor prognosis as reported in genome-wide association studies. Therefore, Ghosh et al. tested radiomics to predict *BAP1* mutation status on ccRCC and found an AUC of 0.71 for features retrieved from the NP phase of CECT scans. Similarly, Kocak et al. aimed to predict the presence of gene *PBRM1* mutations by creating an ANN algorithm and ML-based TA from CMP images; they found that the former outperformed the latter, upholding 95% accuracy and 0.987 AUC for *PBRM1* mutation status. This tumor suppressor gene’s mutation was associated with advanced-stage and higher grade ccRCC, and it was also suggested to influence response rates to immune checkpoint inhibitors [53,54].

Angiogenesis is a largely known pathway in the pathophysiology of RCC, to such an extent that several targeted therapies used in RCC aim to halt its development. Therefore, Yin et al. tested the feasibility to predict tumor vascularity using radiomics features from PET and MRI in a small cohort (*n* = 9). Tumor vascularity, vascular endothelial growth factor (VEGF) expression, and microvascular density were measured from fresh frozen tissue specimens. A correlation between VEGF expression and radiomics features was not found (*p* = 0.539); however, the microvascular density did correlate with radiomic features, and the conjunction of image datasets from both modalities, PET/MRI, displayed the highest correlation (r = 0.639, *p* = 0.044). This proof-of-concept study highlights the possibility to predict pathological findings by radiomics alone, which is of utmost utility when such findings can serve as biomarkers [58].

These data can aid in the construction of nomograms that can exert an influence over clinical decision-making. Thus, some authors paved the path toward personalized medicine by assessing quantitative genomics data as predictors. Park et al. predicted T1 ccRCC aggressiveness with 85% accuracy (AUC: 0.796) by utilizing a model built on deep neural network algorithms and comprising data from *FOXC2*, *PBRM*, and *BAP1* gene and protein expression [56]. Similarly, Li et al. constructed a gene panel including 15 genes (Table 3) and developed an expression-based prediction score which accurately differentiated between low- and high-risk ccRCC and predicted three-year overall survival (AUC: 0.784). In fact, the authors created a nomogram which included clinical and biochemical data, and which may prove to be a clinically indispensable tool after further validation is obtained [55]. Azuaje et al. conducted a study where data from proteomics, gene expression, and DL-based TA of histology samples were examined to discriminate between ccRCC samples and normal tissue. Interestingly, the image-based data correlated with that of proteomics, and the biological processes and pathways of the proteins involved correlated with extracellular organization activities and immune response. Furthermore, complementary RNA (cRNA) expression and gene expression also strongly correlated with image-based data (*r* = 0.76). Despite no radiological TA being performed in these studies, the relevant information obtained can be applied to further develop prediction scores comprising quantitative image data [57].

### 3.5. Disease Progression and Patient Outcome Prediction

The timely identification of patients who are at risk of developing worse outcomes is paramount for treatment clinical decision-making. Furthermore, predicting responses to targeted therapies or resistance profiles would lead to personalized medical algorithms, saving time and resources while avoiding adverse effects of unfruitful therapies, thereby also improving survival rates and RCC patients’ quality of life. Recently, investigators explored these possibilities, and their findings are presented in Table 4.

Antunes et al. reported a pilot study on predicting the response to sunitinib treatment in two patients with metastatic RCC. The authors sought to evaluate the feature differences between test and retest images retrieved from multiphasic MRI and PET scans using fluorothymidine as radiotracer at baseline and three weeks after administering sunitinib. The changes in image texture features were analyzed with ROIs from tumor and normal tissue images to assess any changes in both. Overall, a low variability between test and retest images for both modalities was found. Moreover, 63% difference was found in the top-ranked texture features of tumor regions from pretreatment images and after three weeks of sunitinib treatment, whereas only a 17% difference was found in the normal tissue regions. The authors concluded that MRI and PET texture features can detect early structural and functional changes of treatment response. Naturally, further studies with larger population are warranted [59].

Data extracted from RNA sequencing (RNA-seq) and gene expression profiles were processed and classified using ML-based methods by Singh et al. to discriminate patients with early stages (I–II) and late stages (III–IV) of pRCC. The principal component analysis solely based on gene expression accurately discriminated between normal and tumor samples and between early and late stages. Furthermore, the different algorithms used for most selected features yielded satisfactory accuracy rates, ranging between 82.5% and 88%, along with a precision and recall AUC of 0.69–0.79 and correlation coefficient of 0.60–0.68. Tracing the biological pathways and protein interaction revealed that most expression differences were in processes related to microtubules, chromosomes, and the cell cycle. Interestingly, late stages of pRCC had higher expression of kinetochore and centromere proteins, which are well-known alterations and distinctive neoplastic features leading to chromosome instability [60]. Tabibu et al. classified a TCGA (The Cancer Genome Atlas) dataset into high- and low-risk ccRCC according to a risk index based on tumor and nuclei shape features on histopathology slices constructed by a CNN. After grade discrimination, survival probability was tested, and the high-grade group was associated with a lower survival rate [61]. Clearly, radiomics-based progression prediction in RCC is only in its initial stages; however, if such a goal is reached, it will prove to be a major breakthrough for RCC management.

### 3.6. Limitations/Challenges of Radiomics

Although radiomics is attracting substantial attention in medicine and the field of oncology in general, its real-life implementation in the clinical scenario still faces obstacles. Firstly, the variability in study design, radiomic methods employed, texture features extracted, and recorded endpoints make it difficult to compare any two techniques and to perform quantitative analysis. Secondly, most ML and DL algorithms utilized in these studies were validated with their own dataset; therefore, without external validation, result generalizability and reproducibility cannot be applied to other datasets and populations [62]. Thirdly, repeatability, reproducibility, sample size, statistical power, and standardization are still vital factors to be considered in future investigations [63]. Fourthly, gene-based changes may result in significantly different radiomics features which are not consistent across all imaging phases [64]. Therefore, the link between image tumor properties and tumor biology is not straightforward, correlation does not imply causation, and a statistical relationship between radiomics and genetic footprints or prognosis should be established [65]. Lastly, if dataset heterogeneity is not addressed for the conduction of future studies, bridging the translational leap from an experimental research tool to an essential clinically applicable diagnostic and predictive instrument will be challenging [3].

### 3.7. Future Perspectives

The focus of future research should be constructing larger unified medical registries and databases to further enhance the accuracy of radiomics techniques. The use of improved algorithms should not be limited to large computer analyzing centers but extended to mobile devices and access by cloud services ensuring the protection of users’ personal data. For individualized AI-based software to function with robotic platforms across operating rooms worldwide and take intraoperative decisions in real time, dedicated transnational regulatory approvals will have to be accorded. Additionally, there are major concerns regarding the reliability of diagnoses coming from algorithms and the role of programming biases interfering with patient management. Clearly, human intelligence will continue being the cornerstone in shaping future AI advances to ensure that these systems prioritize patient care and to develop mechanisms that prevent undesired use.

## 4. Conclusions

By further including data and making models more robust, the predictive precision of radiomics will continue contributing toward personalized medicine. Instant predictive analytics can be obtained from large semi-automated patient datasets and electronic medical records to be used for delivering insights into various disease processes. The diagnostic accuracy, however, is highly dependent on the quality of data and its efficient integration with different sources to generalize it. Shared decision-making will certainly not be replaced by these methods, although they may serve as an important accessory to the information patients obtain from traditional medical care. Undoubtedly, we are beginning a new era in medicine, and the future applications that radiomics may bring are limitless.

## Figures and Tables

**Table 1:** Summary of studies applying radiomics for renal tumor differentiation.

Author	Imaging	Patients	Mean Age	Mean Lesion Diameter (cm)	Radiomics Method	Texture Features	Predicted Outcome Accuracy
**Benign vs. Malignant Tumor Differentiation**							
Kunapuli et al. 2018 [17]	Four-phasic CECT	150 patients	NA	NA	11 ML-based classifiers	40 features extracted per phase from all patients (Gray-level histogram, GLCM, GLDM)	***Decision support tool for renal mass classification***
		70 ccRCC					
		20 pRCC					
		10 chRCC					RFGB methods showed the best performance for dealing with class imbalance, accuracy (0.83) and AUC (0.83) from all 11 methods tested.
		20 AMLvwf					
		30 oncocytoma					
Sun et al. 2019 [15]	Three-phasic CECT	290 lesions			ML-based SVM classifiers	35 features for ccRCC vs. pRCC, 22 features for ccRCC vs. AMLwvf and oncocytoma, 11 features for pRCC/chRCC vs. AMLwvf/oncocytoma	***Radiomics ML models for benign*** **vs. *malignant tumor differentiation and comparison with expert radiologists***
		190 ccRCC	59 (23–85)	4.00			
		26 pRCC	54 (19–76)	4.16			
		38 chRCC	51 (24–83)	4.61			Radiologists performance were as follows: sensitivity 73.7–96.8% and specificity of 48.4–71.9% for differentiating ccRCCs from pRCCs and chrRCCs; sensitivity of 73.7–96.8% and specificity of 52.8–88.9% for differentiating ccRCCs from AMLwvf and oncocytomas; and sensitivity of 28.1–60.9% and specificity of 75.0–88.9% for differentiating pRCCs and chrRCCs from AMLwvf and oncocytomas. Radiomic ML model yielded sensitivity of 90.0%, 86.3%, and 73.4% and specificity of 89.1%, 83.3%, and 91.7%, respectively.
		26 AMLwvf	47 (24–68)	2.99			
		10 oncocytoma	42 (28–56)	3.95			
Xi et al. 2020 [19]	MRI T_2_ weighted and T_1_ post contrast	1162 renal lesions	NA	NA	Ensemble deep learning model	Images randomly divided into training set of 816 lesions with 408,000 augmented images, validation set of 234 lesions, and test set of 112 lesions.	***Deep learning model distinguishing benign tumors from RCC***
		655 malignant					
		507 benign					Compared to four expert radiologists, the ensemble DL model had higher test accuracy (0.70 vs. 0.60, *p* = 0.053), sensitivity (0.92 vs. 0.80, *p* = 0.017), and specificity (0.41 vs. 0.35, *p* = 0.450).
Erdim et al. 2020 [18]	Two-phased CECT	63 malignant masses	57.2 ± 12.6	5.84 ± 3.3	Eight ML-based algorithms for model development	198 features from unenhanced phase and 24 from CMP	***ML prediction of benign and malignant renal masses***
		25 ccRCC					The accuracy and AUC were 90.5% and 0.915, respectively. After eliminating the highly collinear features from the analysis, the accuracy and AUC values slightly increased to 91.7% and 0.916, respectively.
		23 pRCC					
		15 chRCC					
		21 benign	54.9 ± 15.5	3.6 ± 1.4			
		11 AMLwvf					
		10 oncocytoma					
**Angiomyolipoma without visible fat (AMLwvf) vs. RCC subtypes**							
Yan et al. 2015 [20]	Three-phasic CECT	Pathologically proven:			ANN classifier	Image histogram, gradient, GLRL matrix, autoregressive model and wavelet transform	***Differentiation between AMLwvf, ccRCC and pRCC***
		18 AML	44.5 (26–61)	2.85 (range, 0.8–5.1)			Excellent classification results (0% to 9.3% error) were obtained for all three groups, independently of CT phase used. Unenhanced phase showed better trend for classification.
		18 ccRCC	53.9 (36–79)	3.3 (range, 1.5–4.9)			
		14 pRCC	57.6 (34–77)	3.3 (range, 1.4–5.1)			
Feng et al. 2018 [21]	Three-phasic CECT	58 SRM patients:			ML-based SMV classifier for quantitative texture analysis	Image histogram and GLCM	***Differentiating AMLwvf from ccRCC***
		41 ccRCC	56.2 ± 12.3	3.2 ± 0.7			SMV classifier discriminated between AMLwvf and ccRCC with accuracy, sensitivity, specificity and AUC of 93.9%, 87.8%, 100% and 0.955, respectively.
		17 AMLwvf	48.7 ± 10.8	2.8 ± 0.9			
Lee et al. 2018 [22]	CECT	41 ccRCC	NA	NA	Deep feature classification with CNN and ML-classifiers	- Hand-crafted texture and shape features	***Deep feature classification of AMLwvf and ccRCC***
		39 AMLwvf				- Deep feature extraction	Improved texture features enhance AMLwvf and ccRCC differentiation.
Cui et al. 2019 [23]	Three-phasic CECT	171 pathologically proven renal masses		NA	ML-based SMV established differentiation classifiers	Shape, GLCM, GLRL matrix, gray-level-size zone matrix, gray-tone difference matrix, and gray-level-dependence matrix	***Differentiation of AMLwvf from all RCC subtypes***
		82 ccRCC	55.3 ± 11.6				Differentiating AML from all-RCC (AUC = 0.96) and ccRCC (AUC = 0.97) was higher than AML from non-ccRCC (AUC = 0.89). Radiologists´ interpretation achieved lower performance differentiating AML from all-RCC (AUC= 0.067), ccRCC (AUC = 0.68), and non-ccRCC (AUC = 0.64).
		22 pRCC	49.3 ± 12.9				
		26 chRCC	55.0 ± 11.8				
		41 AMLwvf	48.6 ± 12.9				
Yang et al. 2019 [24]	Four-phasic CECT	163 SRM patients		(median, IQR)	ML-based SMV, LR and RF classifiers	Extracted features: 1. shape, 2. histogram analysis, and 3. texture features	***Differentiation of AMLwvf from RCC subtypes***
		95 ccRCC					Features extracted from unenhanced phase are sufficient to generate accurate differentiation between AMLwvf and RCC using ML-based classification model. Two models achieved classification AUC of 0.90.
		10 pRCC	52.9 ± 13.1	2.9 (2.5, 3.3)			
		13 chRCC					
		45 AMLwvf	48.6 ± 13.7	2.5 (2.1, 3.3)			
Nie et al. 2019 [25]	Four-phasic CECT	63 ccRCC	58.6 ± 11.5	2.2 (0.8–8.8)	Radiomics feature extraction for signature and nomogram	- Fourteen texture features built radiomics signature	***Differentiating ccRCC from AMLwvf***
		36 AMLwvf	50.1 ± 8.3	2.7 (1.3–6.2)		- Rad-score/clinical factors for nomogram	Decision curve analysis demonstrated the nomogram outperformed the clinical factors model and radiomics signature in terms of clinical usefulness.
Ma et al. 2020 [26]	Three-phasic CECT	62 ccRCC	57.9 ± 10.8	3.7 ± 1.6	Four radiomics logistic classifiers	Radiomics feature parameters: Histogram, texture, form factor, GLCM and RLM	***Differentiating ccRCC from AMLwvf***
		22 AMLwvf	50.5 ± 12.8	3.2 ± 0.9			Whole-tumor radiomics-based CT analysis was superior to conventional CT analysis. Cyst degeneration, pseudocapsule, and sum rad-score were the most significant factors. Unenhanced phase radiomics showed higher AUC than CMP and NP groups.
**Oncocytoma vs. RCC Subtypes**							
Yu et al. 2017 [27]	Two-phasic CECT	119 RCC patients	NA	NA	CT TA with ML-based SVM classifier	43 texture features extracted from renal tumor segments: 14 histogram-based, 5 GLCM, 11 GLRL, 4 GLGM, and 9 Laws’ features.	***Oncocytoma*** **vs. *RCC subtypes***
							Excellent tumor discriminators were identified with AUC of 0.91 and 0.93 (*p* < 0.0001) respectively for differentiating ccRCC from oncocytoma. AUC of 0.99 (*p* < 0.0001) for differentiating pRCC from oncocytoma and an AUC of 0.92 for differentiating oncocytoma from other tumors. The ability of ML to distinguish ccRCC from other tumors and pRCC from other tumors showed AUC of 0.91 and 0.92, respectively.
Coy et al. 2019 [28]	Four-phase CECT	128 ccRCC	62 (22–91)	3.8 (0.8–14.6)	ANN trained with 4000 iterations	ML-based texture extraction	***Oncocytoma*** **vs. *ccRCC***
		51 oncocytoma	69 (38–87)	3.9 (1.0–13.1)			Excretory phase of entire tumor volume achieved highest 74.4% accuracy, 85.8% sensitivity and 80.1% PPV. When combined with tumor mid-slices of all phases then PPV was 82.5%.
Li et al. 2019 [29]	Four-phase CECT	44 chRCC	50.8 (22–79)	NA	Five ML-based classifiers (kNN, SVM, RF, LR, MLP)	- Intensity statistics: peak value, mean value and variance	***Chromophobe RCC*** **vs. *oncocytoma***
						- Shape features: Volume, surface area, spherical value	All five classifiers had good diagnostic performance, with AUC values greater than 0.85. SVM classifier showed the highest diagnostic accuracy with 0.945.
		17 oncocytoma	54.9 (35–79)			- Texture features: GLRL and size matrices	Accurate preoperative differential diagnosis of chRCC and oncocytoma can be facilitated by a combination of CT enhanced quantitative features and ML.
Baghdadi et al. 2020 [30]	Three-phasic CECT	212 renal masses from 192 patients	NA	NA	CNNs deep-learning structure based on TensorFlow	Semi-automated tumor-to-cortex peak early-phase enhancement ratio (PEER)	***Chromophobe RCC*** **vs. *oncocytoma***
							PEER evaluation achieved 95% accuracy in tumor type classification (100% sensitivity and 89% specificity) compared to the final pathology results.
**RCC Subtype Differentiation**							
Li et al. 2018 [31]	Three-phasic CECT	Training: - 170 patients	58.5 (21–84)	NA	ML-based RF classifier	For each tumor 156 texture features extracted from triphasic CECT	***Differentiation of clear cell and non-clear cell RCC and radiomics link to VHL gene mutation***
		Validation: - 85 patients	58.9 (33–81)				
		255 in total					
		118 ccRCC					Eight all-relevant features from CMP were selected. Model showed AUC of 0.95 and an accuracy of 92.9% in the validation cohort. Five out of eight all-relevant features were significantly associated with VHL mutation.
		36 pRCC					
		31 chRCC					
Kocak et al. 2018 [32]	Three-phasic CECT	68 RCC patients	5.9 (3.3–8.1)	5.9 (2.0–12.3)	ANN and ML-based SVM classifiers	275 texture features extracted:	***Distinguishing the three main RCC subtypes***
		48 ccRCC					ANN discrimination of non-ccRCC from ccRCC subtypes with an external validation accuracy, sensitivity, and specificity of 84.6%, 69.2%, and 100%, respectively.
		13 pRCC					SVM discrimination of pRCC from other RCC subtypes with an external validation accuracy, sensitivity, and specificity of 69.2%, 71.4%, and 100%, respectively.
		7 chRCC					
Yin et al. 2018 [33]	PET/MRI	23 specimens from 8 primary ccRCC	NA	NA	Sparse and generalized partial least squares discriminant analysis	168 radiomics features for each tumor	***ccRCC molecular subtype prediction***
							The correct classification rate (CCR) for molecular subtype classification using only radiomics features was 86.96%. When combining messenger RNA (mRNA), microvascular density, and clinical parameters from each specimen with radiomics features the best CCR was 95.65%.
Han et al. 2019 [34]	Three-phasic CECT	169 patients	NA	NA	GoogLeNet CNN	ROI selection in each phase image	***Distinguishing the three main RCC subtypes***
		57 ccRCC					
		56 pRCC					When compared to a biopsy-proven dataset, CNN showed 0.85 accuracy, 0.64–0.98 sensitivity, 0.83–0.93 specificity, and 0.90 AUC.
		56 chRCC					

ML-based classifiers refer to the insertion of a new observation into the appropriate category among others that were based on trained datasets of known observations. Support vector machines (SVM) are supervised learning methods for classification that learn the optimal difference between features of each class. Random forest (RF) is a supervised learning method for a classification that is based on decision trees. AMLwvf, angiomyolipoma without visible fat; ANN, artificial neural network; AUC, area under curve; ccRCC, clear cell renal cell carcinoma; CECT, contrast-enhanced computed tomography; chRCC, chromophobe RCC; CMP, corticomedullar phase; CNN, convoluted neural network; DL, deep learning; GLCM, gray-level co-occurrence matrix; GLGM, gray-level gradient matrix; GLRL, gray-level run-length; LR, logistic regression; ML, machine learning; MRI, magnetic resonance imaging; NA, not available; NP, nephrographic phase; PET, positron-emission tomography; PPV, positive predictive value; pRCC, papillary RCC; RF, random forest; RFGB, relational functional grading boosting; ROI, regions of interest; SMV, support vector machine; SRM, small renal mass; TA, texture analysis.

**Table 2:** Summary of studies applying radiomics to predict nuclear grade in ccRCC.

Author	Imaging	Patients	Mean Age	Mean Lesion Diameter (cm)	Method	Texture Features	Predicted Outcome Accuracy
Bektas et al. 2018 [42]	Single-pase CECT	23 high-grade	59 (35–81)	5.0 (range 1.6–14.5)	ML-based SMV, MLP, naïve Bayes, k-nearest neighbors, and random forest classifiers for quantitative two-dimensional (2D) TA	Histogram, gradient, GRLM, and autoregressive model	***High-grade (Fuhrman 3–4) tumor detection***
		31 low-grade					SMV model predicted high-grade pathology with 85.1% overall accuracy, 91.3% sensitivity, 80.6% specificity, and AUC of 0.860.
Ding et al. 2018 [44]	Three-phasic CECT	Training: 74 low-grade	59.5 (50–65)	NA	ML-based LASSO to select features and build a texture-score	Histogram, GLCOM, and GRLM	***Detection of high-grade (Fuhrman 3–4) and prediction models***
		40 high-grade	62 (52–68)				Training cohort
		Validation: 71 low-grade	58 (52–65)				Texture-score AUC 0.843 (0.765–0.920). Non-TA features (round mass, diameter, artery tumor, relative tumor enhancement value) were compared to TA features and round mass was similar (AUC: 0.723 (0.632–0.803)). Prediction model including both texture and non-texture features did not outperform that including solely TA features in both cohorts.
		21 high-grade	59 (47–64)				
Shu et al. 2018 [45]	Three-phasic CECT	161 low-grade	55.8 ± 10.7	4.8 ± 1.6	LASSO for feature selection. Models built by LR	First-order statistics, shape, GLCOM, GRLM, and gray-level size zone matrix.	***High-grade (Fuhrman 3–4) tumor prediction***
		99 high-grade	59.3 ± 10.8	6.3 ± 2.1			Three models were created using features from CMP, NP, or CMP + NP. CMP model’s accuracy was 71.9%, AUC 0.766 (0.709–0.816), sensitivity 0.602, and specificity 0.838; NP model’s accuracy was 73.8%, AUC 0.818 (0.765–0.838), sensitivity 0.693, and specificity 0.838; and CMP + NP model’s accuracy was 77.7%, AUC 0.822 (0.769–0.866), sensitivity 0.677, and specificity 0.839. The CMP + NP model’s AUC was significantly higher than that of CMP alone and all other AUCs were similar between them.
Gill et al. 2019 [46]	Four-phasic CECT	54 low-grade	61.5	3.40 ± 1.80	Radiomics panel of tissue characterization	Histogram analysis, GLCOM, gray-level difference matrix, 2D Fourier-transform analysis, and spectral analysis	***Differentiating juxtatumoral perinephric fat of high-grade (ISUP 3–4)*** **vs. *low-grade (ISUP 1–2)***
		30 high-grade (ISUP)	61.7	4.33 ± 2.24			All TA methods but gray-level difference matrix showed differences and increased heterogeneity index in high-grade juxtatumoral perinephric fat.The measure of correlation coefficient form GLCOM had the best accuracy (AUC 0.746 (0.63–0.86)).
Goyal et al. 2019 [47]	Multi-phasic MRI	19 low-grade	50.3 (including 5 non-ccRCC)	6.63 ± 3.2 (including 5 non-ccRCC)	ML-based TexRAD arranging according to size in SSF	Filtration histogram.	***High-grade (Fuhrman 3–4) tumor detection***
		10 high-grade					The best performance was found in Entropy (at SSF 6 on diffusion-weighted image) AUC: 0823 (0.618–1.0), mean (at SSF 3 on CMP) AUC: 0.889 (0.655–1.9), and mean of positive pixels (at SSF 5 on NP) AUC: 0.870 (0.712–1.0)
He et al. 2019 [48]	Three-phasic CECT	136 low-grade	57.3 ± 12.9	NA	ML-based on ANN fed with radiomics signatures prediction models	Gray-level histogram, GLCOM, GRLM, histogram of oriented gradient, wavelet transformations, and autoregressive models	*** Prediction accuracy of high-grade (ISUP 3–4) tumors by 5 TA-based models ***
		91 high-grade (ISUP)					Five models based on features with the best performance had a predictive mean value of 92.46% ± 1.83%. The top-ranked model was a combination of minimum mean squared error of conventional image features and CMP phase (94.06% ± 1.14%)
Kocak et al. 2019 [43]	NECT	25 low-grade	62	7.59 (range 2.5–16.4)	ANN and binary LR with and without SMOTE	First order, GLDM, GLCOM, GRLM, gray-level size zone matrix, neighboring gray-tone difference matrix, and wavelet-based features	*** High-grade (Fuhrman 3–4) tumor detection ***
		56 high-grade (Fuhrman)					The ANN algorithm (based on 5 TA features) outperformed that of logistic regression (based on 6 features). ANN algorithm detected 81.5% of high-grade tumors accurately (AUC: 0.714).
Lin et al. 2019 [49]	Three-phasic CECT	189 low-grade	54.9 ± 11.9	NA	ML-based CatBoost	First-order, shape, GLCOM, GRLM, gray-level size zone matrix, and GLDM features.	*** High-grade (Fuhrman 3–4) tumor detection ***
		43 high-grade (Fuhrman)	53.1 ± 12.6				The ML model based on three-phase CT images detected high-grade tumors with an AUC 0.87, outperforming those based on single-phase images.
Shu et al. 2019 [50]	Three-phasic CECT	164 low-grade	57.3 ± 10.9	4.7 ± 1.5	LASSO for feature selection. The k-nearest neighbor, LR, MLP, random forest, and SVM as ML-based classifiers	First-order statistics, shape, GLCOM, GRLM, and gray-level size zone matrix.	***High-grade (ISUP 3–4) tumor prediction***
		107 high-grade (ISUP)		6.2 ± 2.0			The best model was achieved by the combined classifier (CMP + NP features) with 91.7%–93.5% accuracy and an AUC of 0.96–0.98 in the validation cohort compared to the training cohort with 86.5%–90.8% accuracy and an AUC of 0.95–0.97.
Sun et al. 2019 [51]	Three-phasic CECT	155 low-grade	53 (47–62)	NA	ML-based SMV. Variant selection and LASSO for feature selection	First-order statistics, shape and size, GRLM, GLCOM, and higher-order statistics (from wavelet transformation)	***High-grade (ISUP 3–4) tumor prediction***
		72 high-grade (ISUP)	57 (51–65)				A model combining features of both phases (CMP and NP) with SMV classifier achieved best performance in the training and validation datasets, with an AUC of 0.88 (0.77–0.95; sensitivity 0.85 and specificity 0.89) and 0.91 (0.65–0.99, sensitivity 0.83 and specificity 0.89), respectively.
Cui et al. 2020 [52]	Three-phasic CECT and multiphasic MRI	Internal cohort: 347 CTE, 93 MRI, 284 low-grade	44.4 (28–88)	NA	ML-based CatBoost	First order features, shape features, GLCOM, GLDM, gray-tone difference matrix, GRLM, gray-level size-zone matrix	***Comparison between CECT- and MR-based high-grade (ISUP 3–4) prediction***
		156 high-grade	57.4 (24–85)				MRI ML-TA accuracy did not outperform that of CT either in the internal (79% vs. 73%) or in the external (69% vs. 74%) cohorts’ datasets.
		External cohort:20 CECT + MRI, 10 low-grade	54.3 (38–70)				***High-grade accuracy prediction and external validation***
		10 high-grade (ISUP)	60.8 (42–76)				CECT and MRI multiphase TA improved accuracy prediction 2–10% compared to single-phase. Similar results between cohort datasets were reported.

Classifiers in ML refer to the insertion of a new observation into the appropriate category among others that were based on trained datasets of known observations. Support vector machines are supervised learning methods for classification that learn the optimal difference between features of each class. Random forest is a supervised learning method for classification that is based on decision trees. CatBoost is a gradient boosting decision library based on decision trees. k-Nearest neighbor in non-parametric statistic algorithm for classification. SMOTE is synthetic minority over-sampling technique and serves as a classification model. ANN, artificial neural network; ccRCC, clear cell renal cell carcinoma; CECT, contrast enhanced compute tomography; CMP, corticomedullary phase; CT, computed tomography; GLCOM, gray-level co-occurrence matrix; GLDM, gray-level dependence matrix; GRLM, gray-level run-length matrix; ISUP, International Society of Urological Pathology; LASSO, the least absolute shrinkage and selection operator; LR logistic regression; ML, machine learning; MLP, multilayer perceptron; MRI, magnetic resonance imaging; NCCT: non-contrast enhanced compute tomography; NP, nephrographic phase; ROC, receiver operating characteristics; SMV, support vector machine; SSF, spatial scaling factors; TA, texture analysis.

**Table 3:** Summary of studies applying radiomics and gene expression-based models to identify molecular biomarkers.

Author	Source	Patients	Mean Age	Method	Biomarker(s)	Texture Features	Predicted Outcome Accuracy
Ghosh et al. 2015 [53]	Four-phasic CECT	14 ccRCC *BAP1*-mutant and 64 non-mutant	NA	Image-genomics pipeline. Texture features from 3D-tumor images. Random forest classifiers. Data from TCGA	*BAP1*	Histogram, Haralick, GRLM, GLCOM, mean gray-level intensity	***Prediction of BAP1 mutation status by ML-based 3D-TA***
							Best rated model was that based on NP, with an AUC of 0.71.
Kocak et al. 2018 [54]	CMP of CECT	16 ccRCC mutant *PBRM1* and 29 non-mutant. (12 low- and 33 high-grade)	60	ANN and ML-based TA from images using random forest classifiers. Data from TCGA	*PBRM1*	Firs-order, GLDM, GLCOM, GRLM, gray-level size zone matrix, neighbor gray-tone difference matrix, and wavelet-based features.	***Prediction of PBRM1 mutation status by ANN and ML-based TA***
							ANN algorithm’s accuracy: 88.2%; AUC: 0.925. The random forest model’s accuracy: 95%; AUC: 0.987.
Li et al. 2018 [55]	Genes expression panel	533 ccRCC	NA	Genes whose expression was associated with OS were selected and downsized by RF variable selection, then were categorized as high/low risk groups according mean genes’ expression	*COL7A1, ARFGAP1, BRD9, MC1R, ATP13A1, POFUT2, OTOF, ANAPC5, CDCA3, IL20RB, CDC7, FBXO3, ZIC2, KL* and *CCDC137*	None	***Correlation between genes’ expression-based risk score and OS***
							Low-risk group had better prognosis and recurrence-free survival. AUC for the risk score and 3-year OS was 0.784
Park et al. 2019 [56]	cDNA extracted from paraffin-embedded tumor tissue	40 aggressive t1 ccRCC	58.0 ± 11.3	Complementary DNA (cDNA) extracted from paraffin-embedded tumor samples. Genes whose expression was different in aggressiveness were IHQ stained. DNN and LR model algorithms	*FOXC2, CLIP4, PBRM1, BAP1, SETD2,* and *KDM5C*	None	***Association of biomarkers with aggressiveness***
		137 non-aggressive T1 ccRCC	58.7 ± 11.8				Lower *FOXC2*, *PBRM1*, and *BAP1* expression was associated with aggressiveness. DNN model’s accuracy based on gene expression was: 0.537 (AUC: 0.736), and LR: 0.555 (AUC: 0.651). Accuracy was significantly increased by adding IHQ data: DNN 0.852 (AUC: 0.796); LR: 0.759 (AUC: 0.760)
Azuaje et al. 2019 [57]	Histopathology and proteomics	110 proteomics 524 histology		Proteomics- and histology-based ML models. RF for proteomics data and DL for histology images data	NA	Raw pixel intensity data from thumbnails of whole slides	***Model*** ***accuracy detecting*** ***ccRCC***
							Proteomic-based model’s accuracy 0.98, sensitivity and specificity of 0.97 and 0.99, respectively. Histology-based model’s prediction 0.95, sensitivity and specificity of 1 and 0.93, respectively.
Yin et al. 2017 [58]	PET-FDG/MRI	9 ccRCC	NA	Analysis of sparse canonical correlation	VEGF expression and MVD	SUV, spatiotemporal association and texture features	***Correlation between vascularity and radiomics features***
							PET/MRI combination had the strongest correlation to MVD. No association of VEGF expression and radiomic features.

Classifiers in ML refer to the insertion of a new observation into the appropriate category among others that were based on trained datasets of known observations. Random forest is a supervised learning method for classification that is based on decision trees. ANN, artificial neural network; AUC, area under the curve; ccRCC, clear cell renal cell carcinoma; CECT, contrast-enhanced compute tomography; CMP, corticomedullary phase; CT, computed tomography; DNN, deep neural network; FDG, fluorodeoxyglucose radiotracer; GLCOM, gray-level co-occurrence matrix; GLDM, gray-level dependence matrix; GRLM, gray-level run-length matrix; IHQ, immunohistochemistry; LR logistic regression; LR, logistic regression; ML, machine learning; MVD, microvascular density; NP, nephrographic phase; OS, overall survival; ROI, regions of interest; SUV, standardized uptake value; TA, texture analysis; TCGA; The Cancer Genome Atlas; VEGF, vascular endothelial growth factor.

**Table 4:** Summary of studies applying radiomics and gene expression-based models to predict progression in ccRCC.

Author	Source	Patients	Method	Features	Predicted Outcome Accuracy
Antunes et al. 2016 [59]	PET -FLT and multiphase MRI	2 patients with advanced ccRCC	Radiomics analysis of test/retest images	First- and second-order statistical features, ADC Haralick, entropy and difference average features.	***Detection of changes in test/retest images after 3 weeks of sunitinib in metastatic RCC*** **.**
					Low variability between test/re-test images. SUV, ADC energy, and T_2_-weigthed average differences were found, and these might be able to detect early structural and functional changes in response to treatment.
Singh et al. 2018 [60]	RNA sequencing and microarray dataset form from GDC	32 normal 289 pRCC:	ML-based algorithms with random forest, naïve Bayes, SVM, KNN, and shrunken centroid classifier	Upregulated and downregulated genes.	***Prediction of progression between early (I–II) and late (III–IV) pRCC stages***
		172 stage I			Gene expression alone discriminated between normal tissue and tumor, and between early and late stage samples by means of PCA. For most features selected, accuracy ranged from 82.5–88%, PR-AUC 0.69–0.79, and MCC 0.60–0-68
		22 stage II			
		51 stage III			
		15 stage IV			
Tabibu et al. 2019 [61]	Histopathology slides from TCGA	1027 ccRCC	CNN model to develop a risk index using LASSO	Tumor and nuclei shape features (area, perimeter, etc.) from tumor region	***Survival probability between high and low grade***
		303 pRCC			Samples were dichotomized in high- and low grade according the risk score constructed form tumor and nuclei features. High-grade group was associated with a lower survival rate (*p* = 3.86 × 10^−6^)
		254 chRCC			

Classifiers in ML refer to the insertion of a new observation into the appropriate category among others that were based on trained datasets of known observations. Support vector machines are supervised learning methods for classification that learn the optimal difference between features of each class. Random forest is a supervised learning method for classification that is based on decision trees. ADC, apparent diffusion coefficient; AUC, area under the receiving operator characteristics curve; ccRCC, clear cell renal cell carcinoma; chRCC, chromophobe renal cell carcinoma; CNN, convolutional neural network; FLT, fluorothymidine radiotracer; GDC, Genomics Data Commons data portal; KNN, k-nearest neighbor; LASSO, the least absolute shrinkage and selection operator; MCC, Matthews correlation coefficient; MRI, magnetic resonance imaging; PCA, principal component analysis; PET, positron-emission tomography; PR-AUC, precision and recall area under the curve; pRCC, papillary renal cell carcinoma; SMV, support vector machine; SUV, standard uptake value; TCGA, The Cancer Genome Atlas.

## References

[1] Ferlay J., Colombet M., Soerjomataram I., Mathers C., Parkin D.M., Piñeros M., et Znaor A., Bray F. (2019). Estimating the global cancer incidence and mortality in 2018: GLOBOCAN sources and methods. Int. J. Cancer.

[2] Siegel R.L., Miller K.D., Jemal A. (2019). Cancer statistics, 2019. CA Cancer J. Clin..

[3] Ursprung S., Beer L., Bruining A., Woitek R., Stewart G.D., Gallagher F.A., Sala E. (2020). Radiomics of computed tomography and magnetic resonance imaging in renal cell carcinoma—a systematic review and meta-analysis. Eur. Radiol..

[4] Patel H.D., Johnson M.H., Pierorazio P.M., Sozio S.M., Sharma R., Iyoha E., Bass E.B., Allaf M.E. (2016). Diagnostic accuracy and risks of biopsy in the diagnosis of a renal mass suspicious for localized renal cell carcinoma: Systematic review of the literature. J. Urol..

[5] Marconi L., Dabestani S., Lam T.B., Hofmann F., Stewart F., Norrie J., Bex A., Bensalah K., Canfield S.E., Hora M. (2016). Systematic review and meta-analysis of diagnostic accuracy of percutaneous renal tumour biopsy. Eur. Urol..

[6] Defortescu G., Cornu J.N., Béjar S., Giwerc A., Gobet F., Werquin C., Pfister C., Nouhaud F.X. (2017). Diagnostic performance of contrast-enhanced ultrasonography and magnetic resonance imaging for the assessment of complex renal cysts: A prospective study. Int. J. Urol..

[7] Karlo C.A., Di Paolo P.L., Donati O.F., Russo P., Tickoo S., Hricak H., Akin O. (2013). Renal cell carcinoma: Role of mr imaging in the assessment of muscular venous branch invasion. Radiology.

[8] Hindman N., Ngo L., Genega E.M., Melamed J., Wei J., Braza J.M., Rofsky N.M., Pedrosa I. (2012). Angiomyolipoma with minimal fat: Can it be differentiated from clear cell renal cell carcinoma by using standard MR techniques?. Radiology.

[9] Gillies R.J., Kinahan P.E., Hricak H. (2016). Radiomics Overview Hricak. Radiology.

[10] De Leon A.D., Kapur P., Pedrosa I. (2019). Radiomics in Kidney Cancer: MR Imaging. Magn. Reson. Imaging Clin. N. Am..

[11] Hollingsworth J.M., Miller D.C., Daignault S., Hollenbeck B.K. (2006). Rising incidence of small renal masses: A need to reassess treatment effect. J. Natl. Cancer Inst..

[12] Pierorazio P.M., Johnson M.H., Patel H.D., Sozio S.M., Sharma R., Iyoha E., Bass E.B., Allaf M.E. (2016). Management of Renal Masses and Localized Renal Cancer: Systematic Review and Meta-Analysis. J. Urol..

[13] Patel H.D., Semerjian A., Gupta M., Pavlovich C.P., Johnson M.H., Gorin M.A., Allaf M.E., Pierorazio P.M. (2019). Surgical removal of renal tumors with low metastatic potential based on clinical radiographic size: A systematic review of the literature. Urol. Oncol. Semin. Orig. Investig..

[14] Akdogan B., Gudeloglu A., Inci K., Gunay L.M., Koni A., Ozen H. (2012). Prevalence and predictors of benign lesions in renal masses smaller than 7 cm presumed to be renal cell carcinoma. Clin. Genitourin. Cancer.

[15] Sun X.Y., Feng Q.X., Xu X., Zhang J., Zhu F.P., Yang Y.H., Zhang Y.D. (2020). Radiologic-Radiomic Machine Learning Models for Differentiation of Benign and Malignant Solid Renal Masses: Comparison With Expert-Level Radiologists. Am. J. Roentgenol..

[16] Waterhouse D.J., Fitzpatrick C.R.M., Pogue B.W., O’Connor J.P.B., Bohndiek S.E. (2019). A roadmap for the clinical implementation of optical-imaging biomarkers. Nat. Biomed. Eng..

[17] Kunapuli G., Varghese B.A., Ganapathy P., Desai B., Cen S., Aron M., Gill I., Duddalwar V. (2018). A Decision-Support Tool for Renal Mass Classification. J. Digit. Imaging.

[18] Erdim C., Yardimci A.H., Bektas C.T., Kocak B., Koca S.B., Demir H., Kilickesmez O. (2020). Prediction of Benign and Malignant Solid Renal Masses: Machine Learning-Based CT Texture Analysis. Acad. Radiol..

[19] Xi I.L., Zhao Y., Wang R., Chang M., Purkayastha S., Chang K., Huang R.Y., Silva A.C., Vallieres M., Habibollahi P. (2020). Deep learning to distinguish benign from malignant renal lesions based on routine MR imaging. Clin. Cancer Res..

[20] Yan L., Liu Z., Wang G., Huang Y., Liu Y., Yu Y., Liang C. (2015). Angiomyolipoma with Minimal Fat: Differentiation From Clear Cell Renal Cell Carcinoma and Papillary Renal Cell Carcinoma by Texture Analysis on CT Images. Acad. Radiol..

[21] Feng Z., Rong P., Cao P., Zhou Q., Zhu W., Yan Z., Liu Q., Wang W. (2018). Machine learning-based quantitative texture analysis of CT images of small renal masses: Differentiation of angiomyolipoma without visible fat from renal cell carcinoma. Eur. Radiol..

[22] Lee H., Hong H., Kim J., Jung D.C. (2018). Deep feature classification of angiomyolipoma without visible fat and renal cell carcinoma in abdominal contrast-enhanced CT images with texture image patches and hand-crafted feature concatenation. Med. Phys..

[23] Cui E.M., Lin F., Li Q., Li R.G., Chen X.M., Liu Z.S., Long W.S. (2019). Differentiation of renal angiomyolipoma without visible fat from renal cell carcinoma by machine learning based on whole-tumor computed tomography texture features. Acta Radiol..

[24] Yang R., Wu J., Sun L., Lai S., Xu Y., Liu X., Ma Y., Zhen X. (2020). Radiomics of small renal masses on multiphasic CT: Accuracy of machine learning–based classification models for the differentiation of renal cell carcinoma and angiomyolipoma without visible fat. Eur. Radiol..

[25] Nie P., Yang G., Wang Z., Yan L., Miao W., Hao D., Wu J., Zhao Y., Gong A., Cui J. (2020). A CT-based radiomics nomogram for differentiation of renal angiomyolipoma without visible fat from homogeneous clear cell renal cell carcinoma. Eur. Radiol..

[26] Ma Y., Cao F., Xu X., Ma W. (2020). Can whole-tumor radiomics-based CT analysis better differentiate fat-poor angiomyolipoma from clear cell renal cell caricinoma: Compared with conventional CT analysis?. Abdom. Radiol..

[27] Yu H.S., Scalera J., Khalid M., Touret A.S., Bloch N., Li B., Qureshi M.M., Soto J.A., Anderson S.W. (2017). Texture analysis as a radiomic marker for differentiating renal tumors. Abdom. Radiol..

[28] Coy H., Hsieh K., Wu W., Nagarajan M.B., Young J., Douek M.L., Brown M.S., Scalzo F., Raman S.S. (2019). Deep learning radiomics: The utility of Google TensorFlowTMInception in classifying clear cell renal cell carcinoma oncocytoma on multiphasic, C.T. Abdom. Radiol..

[29] Li Y., Huang X., Xia Y., Long L. (2019). Value of radiomics in differential diagnosis of chromophobe renal cell carcinoma and renal oncocytoma. Abdom. Radiol..

[30] Baghdadi A., Aldhaam N.A., Elsayed A.S., Hussein A.A., Cavuoto L.A., Kauffman E., Guru K.A. (2020). Automated differentiation of benign renal oncocytoma and chromophobe renal cell carcinoma on computed tomography using deep learning. BJU Int..

[31] Li Z.C., Zhai G., Zhang J., Wang Z., Liu G., Wu G.Y., Liang D., Zheng H. (2019). Differentiation of clear cell and non-clear cell renal cell carcinomas by all-relevant radiomics features from multiphase CT: A VHL mutation perspective. Eur. Radiol..

[32] Kocak B., Yardimci A.H., Bektas C.T., Turkcanoglu M.H., Erdim C., Yucetas U., Koca S.B., Kilickesmez O., Kilickesmez N.O. (2018). Textural differences between renal cell carcinoma subtypes: Machine learning-based quantitative computed tomography texture analysis with independent external validation. Eur. J. Radiol..

[33] Yin Q., Hung S.C., Rathmell W.K., Shen L., Wang L., Lin W., Fielding J.R., Khandani A.H., Woods M.E., Milowsky M.I. (2018). Integrative radiomics expression predicts molecular subtypes of primary clear cell renal cell carcinoma. Clin. Radiol..

[34] Han S., Hwang S.I., Lee H.J. (2019). The Classification of Renal Cancer in 3-Phase CT Images Using a Deep Learning Method. J. Digit. Imaging.

[35] Flum A.S., Hamoui N., Said M.A., Yang X.J., Casalino D.D., McGuire B.B., Perry K.T., Nadler R.B. (2016). Update on the Diagnosis and Management of Renal Angiomyolipoma. J. Urol..

[36] Lim R.S., Flood T.A., McInnes M.D.F., Lavallee L.T., Schieda N. (2018). Renal angiomyolipoma without visible fat: Can we make the diagnosis using CT and MRI?. Eur. Radiol..

[37] Van Der Kwast T., Perez-Ordoñez B. (2007). Renal oncocytoma, yet another tumour that does not fit in the dualistic benign/malignant paradigm?. J. Clin. Pathol..

[38] Wu J., Zhu Q., Zhu W., Chen W., Wang S. (2016). Comparative study of CT appearances in renal oncocytoma and chromophobe renal cell carcinoma. Acta Radiol..

[39] Ishigami K., Jones A.R., Dahmoush L., Leite L.V., Pakalniskis M.G., Barloon T.J. (2015). Imaging spectrum of renal oncocytomas: A pictorial review with pathologic correlation. Insights Imaging.

[40] Thurnher M., Putz T., Rahm A., Gander H., Ramoner R., Bartsch G., Höltl L., Falkensammer C. (2008). Renal Cell Carcinoma. Handb. Dendritic Cells.

[41] Wagener N., Edelmann D., Benner A., Zigeuner R., Borgmann H., Wolff I., Krabbe L.M., Musquera M., Dell´Oglio P., Capitanio U. (2017). Outcome of papillary versus clear cell renal cell carcinoma varies significantly in non-metastatic disease. PLoS ONE.

[42] Bektas C.T., Kocak B., Yardimci A.H., Turkcanoglu M.H., Yucetas U., Koca S.B., Erdim C., Kilickesmez O. (2019). Clear Cell Renal Cell Carcinoma: Machine Learning-Based Quantitative Computed Tomography Texture Analysis for Prediction of Fuhrman Nuclear Grade. Eur. Radiol..

[43] Kocak B., Durmaz E.S., Ates E., Kaya O.K., Kilickesmez O. (2019). Unenhanced CT texture analysis of clear cell renal cell carcinomas: A machine learning-based study for predicting histopathologic nuclear grade. Am. J. Roentgenol..

[44] Ding J., Xing Z., Jiang Z., Chen J., Pan L., Qiu J., Xing W. (2018). CT-based radiomic model predicts high grade of clear cell renal cell carcinoma. Eur. J. Radiol..

[45] Shu J., Tang Y., Cui J., Yang R., Meng X., Cai Z., Zhang J., Xu W., Wen D., Yin H. (2018). Clear cell renal cell carcinoma: CT-based radiomics features for the prediction of Fuhrman grade. Eur. J. Radiol..

[46] Gill T.S., Varghese B.A., Hwang D.H., Cen S.Y., Aron M., Aron M., Duddalwar V.A. (2019). Juxtatumoral perinephric fat analysis in clear cell renal cell carcinoma. Abdom. Radiol..

[47] Goyal A., Razik A., Kandasamy D., Seth A., Das P., Ganeshan B., Sharma R. (2019). Role of MR texture analysis in histological subtyping and grading of renal cell carcinoma: A preliminary study. Abdom. Radiol..

[48] He X., Wei Y., Zhang H., Zhang T., Yuan F., Huang Z., Han F., Song B. (2020). Grading of Clear Cell Renal Cell Carcinomas by Using Machine Learning Based on Artificial Neural Networks and Radiomic Signatures Extracted From Multidetector Computed Tomography Images. Acad. Radiol..

[49] Lin F., Cui E.M., Lei Y., Luo L.P. (2019). CT-based machine learning model to predict the Fuhrman nuclear grade of clear cell renal cell carcinoma. Abdom. Radiol..

[50] Shu J., Wen D., Xi Y., Xia Y., Cai Z., Xu W., Meng X., Liu B., Yin H. (2019). Clear cell renal cell carcinoma: Machine learning-based computed tomography radiomics analysis for the prediction of WHO/ISUP grade. Eur. J. Radiol..

[51] Sun X., Liu L., Xu K., Li W., Huo Z., Liu H., Shen T., Pan F., Jiang Y., Zhang M. (2019). Prediction of ISUP grading of clear cell renal cell carcinoma using support vector machine model based on CT images. Medicine (Baltimore).

[52] Cui E., Li Z., Ma C., Li Q., Lei Y., Lan Y., Yu J., Zhou Z., Li R., Long W. (2020). Predicting the ISUP grade of clear cell renal cell carcinoma with multiparametric MR and multiphase CT radiomics. Eur. Radiol..

[53] Ghosh P., Tamboli P., Vikram R., Rao A. (2015). Imaging-genomic pipeline for identifying gene mutations using three-dimensional intra-tumor heterogeneity features. J. Med. Imaging.

[54] Kocak B., Durmaz E.S., Ates E., Ulusan M.B. (2019). Radiogenomics in Clear Cell Renal Cell Carcinoma: Machine Learning-Based High Dimensional Quantitative CT Texture Analysis in Predicting PBRM1 Mutation Status. AJR Am. J. Roentgenol..

[55] Li P., Ren H., Zhang Y., Zhou Z. (2018). Fifteen-gene expression based model predicts the survival of clear cell renal cell carcinoma. Medicine (US).

[56] Park J.S., Lee H.J., Cho N.H., Kim J., Jang W.S., Heo J.E., Ham W.S. (2019). Risk Prediction Tool for Aggressive Tumors in Clinical T1 Stage Clear Cell Renal Cell Carcinoma Using Molecular Biomarkers. Comput. Struct. Biotechnol. J..

[57] Azuaje F., Kim S.-Y., Perez Hernandez D., Dittmar G. (2019). Connecting Histopathology Imaging and Proteomics in Kidney Cancer through Machine Learning. J. Clin. Med..

[58] Yin Q., Hung S.C., Wang L., Lin W., Fielding J.R., Rathmell W.K., Khandani A.H., Woods M.E., Milowsky M.I., Brooks S.A. (2017). Associations between Tumor Vascularity, Vascular Endothelial Growth Factor Expression and PET/MRI Radiomic Signatures in Primary Clear-Cell-Renal-Cell-Carcinoma: Proof-of-Concept Study. Sci. Rep..

[59] Antunes J., Viswanath S., Rusu M., Valls L., Hoimes C., Avril N., Madabhushi A. (2016). Radiomics analysis on FLT-PET/MRI for characterization of early treatment response in renal cell carcinoma: A proof-of-concept study. Transl. Oncol..

[60] Singh N.P., Bapi R.S., Vinod P.K. (2018). Machine learning models to predict the progression from early to late stages of papillary renal cell carcinoma. Comput. Biol. Med..

[61] Tabibu S., Vinod P.K., Jawahar C.V. (2019). Pan-Renal Cell Carcinoma classification and survival prediction from histopathology images using deep learning. Sci. Rep..

[62] Kocak B., Durmaz E.S., Erdim C., Ates E., Kaya O.K., Kilickesmez O. (2020). Radiomics of Renal Masses: Systematic Review of Reproducibility and Validation Strategies. AJR Am. J. Roentgenol..

[63] Avanzo M., Stancanello J., El Naqa I. (2017). Beyond Imaging: The Promise of Radiomics. Phys. Med..

[64] Panth K.M., Leijenaar R.T.H., Carvalho S., Lieuwes N.G., Yaromina A., Dubois L., Lamblin P. (2015). Is there a causal relashionship between genetic changes and radiomics-based image features? An in vivo preclinical experiment with doxycycline inducible GADD34 tumor cells. Radiother. Oncol..

[65] Napel S., Giger M. (2015). Special Section Guest Editorial: Radiomics and Imaging Genomics: Quantitative Imaging for Precision Medicine. J. Med. Imaging.

